# Decreasing incidence of both major histologic subtypes of gastric adenocarcinoma – a population-based study in Sweden

**DOI:** 10.1054/bjoc.2000.1205

**Published:** 2000-07-03

**Authors:** A M Ekström, L-E Hansson, L B Signorello, A Lindgren, R Bergström, O Nyrén

**Affiliations:** Department of Medical Epidemiology, Karolinska Institutet, Stockholm, S-171 77, Sweden; Department of Surgery, Mora Hospital, Mora, S-792 85, Sweden; Department of Epidemiology, Harvard School of Public Health, Boston, MA, 02115, USA; Department of Pathology, Falu Hospital, Falun, S-791 82, Sweden; Department of Statistics, Uppsala University, Uppsala, S-751 20, Sweden

**Keywords:** stomach neoplasms, incidence, histologic type, cardia, *Helicobacter pylori*

## Abstract

While the overall incidence of gastric cancer has fallen, presumably to a large extent in parallel with *Helicobacter pylori* infection, the occurrence of the diffuse histologic type is thought to have remained more stable, questioning the aetiologic role of *H. pylori*. We have analysed the incidence of the intestinal and diffuse types separately, while considering subsite (cardia/non-cardia). With an extensive prospective effort we identified all incident cases of gastric adenocarcinoma (*n* = 1337) in a well-defined Swedish population (1.3 million) 1989–1994. Tumours were uniformly classified histologically and topographically. Subgroup-specific incidence rates were computed and modelled using multivariate logistic regression. Site-specific trends were clearly discrepant. The overall incidence of adenocarcinoma distal to the gastric cardia declined by 9% (95% confidence interval 6–12%) per year, while cardia cancer remained stable. Thus, the feared rise in cardia cancer could not be confirmed despite clear site-specific trend discrepancies. The intestinal type predominated, especially in high-risk areas, while diffuse tumours prevailed among young patients and women. Both main histologic types of gastric adenocarcinoma declined markedly, at similar rapidity, and with no significant trend differences between the intestinal and diffuse types, even after multivariate adjustments. Our results are consistent with an aetiologic role of environmental factors including *H. pylori* also for diffuse-type gastric cancers. © 2000 Cancer Research Campaign


British Journal of Cancer (2000) **83**(3), 391–396
© 2000 Cancer Research Campaign
doi: 10.1054/ bjoc.2000.1205, available online at http://www.idealibrary.com on IDEAL®

### Decreasing incidence of both major histologic subtypes of gastric adenocarcinoma – a population-based study in Sweden

**AM Ekström[1], L-E Hansson[1,2], LB Signorello[1,3], A Lindgren[4], R Bergström[1,5] and O Nyrén[1]**

[1]Department of Medical Epidemiology, Karolinska Institutet, S-171 77 Stockholm, Sweden; [2]Department of Surgery, Mora Hospital, S-792 85 Mora, Sweden; [3]Department of Epidemiology, Harvard School of Public Health, Boston, MA 02115, USA; [4]Department of Pathology, Falu Hospital, S-791 82 Falun, Sweden; [5]Department of Statistics, Uppsala University, S-751 20 Uppsala, Sweden

**Summary** While the overall incidence of gastric cancer has fallen, presumably to a large extent in parallel with *Helicobacter pylori* infection, the occurrence of the diffuse histologic type is thought to have remained more stable, questioning the aetiologic role of *H. pylori*. We have analysed the incidence of the intestinal and diffuse types separately, while considering subsite (cardia/non-cardia). With an extensive prospective effort we identified all incident cases of gastric adenocarcinoma ($n$ = 1337) in a well-defined Swedish population (1.3 million) 1989–1994. Tumours were uniformly classified histologically and topographically. Subgroup-specific incidence rates were computed and modelled using multivariate logistic regression. Site-specific trends were clearly discrepant. The overall incidence of adenocarcinoma distal to the gastric cardia declined by 9% (95% confidence interval 6–12%) per year, while cardia cancer remained stable. Thus, the feared rise in cardia cancer could not be confirmed despite clear site-specific trend discrepancies. The intestinal type predominated, especially in high-risk areas, while diffuse tumours prevailed among young patients and women. Both main histologic types of gastric adenocarcinoma declined markedly, at similar rapidity, and with no significant trend differences between the intestinal and diffuse types, even after multivariate adjustments. Our results are consistent with an aetiologic role of environmental factors including *H. pylori* also for diffuse-type gastric cancers.
© 2000 Cancer Research Campaign

**Keywords:** stomach neoplasms; incidence; histologic type; cardia; *Helicobacter pylori*

Gastric carcinoma continues to claim an increasing number of victims (approximately 900 000 per year) partly due to the ageing of the population (Munoz and Franceschi, 1997), but as we enter the 21st century, the aetiology of the world's second most common cancer (Parkin et al, 1993) remains relatively poorly understood. Risk factors for the different subtypes, both according to tumour histology (intestinal/diffuse) (Lauren, 1965) and site (cardia/non-cardia), need to be identified.

The remarkable global decline in gastric carcinoma incidence has mainly been attributed to intestinal type (Correa and Shiao, 1994; Lauren and Nevalainen, 1993; Munoz and Asvall, 1971) although firm consensus is lacking. Its variation over time and across populations (Coleman et al, 1993) is presumed to have a predominantly environmental aetiology. In contrast, the diffuse subtype is usually postulated to be more genetically predetermined (Correa and Shiao, 1994). Reports of a disparate incidence trend for cardia tumours (Blot et al, 1991; Powell and McConkey, 1992) strongly suggest that this subsite has a distinct aetiology.

The concurrently falling prevalence of infection with *Helicobacter pylori* (*H. pylori*) (Haruma et al, 1997; Parsonnet et al, 1992; Roosendaal et al, 1997; Xia and Talley, 1997), suggests that its association with non-cardia gastric carcinoma is confined to the intestinal subtype. This hypothesis is in line with Correa's model of *H. pylori*-induced carcinogenesis (Correa, 1995). A stable incidence of the diffuse subtype would contradict an important role of *H. pylori*.

We conducted a prospective population-based study over 6 years, with an extensive search for all incident gastric cancer cases in well-defined populations and a strictly uniform classification of tumour site and histologic type. Our aim was to analyse the incidence of the intestinal and diffuse types separately, while taking subsite into account. Sweden, with a two-fold variation in gastric cancer risk within the country and declining gastric cancer rates, is a particularly suitable environment for studying these questions.

### MATERIALS AND METHODS

### Subjects

All new cases of gastric adenocarcinoma diagnosed before death, from 1989 through 1994, occurring in two northern high-risk counties (Norrbotten, Västerbotten) and three southern low-average-risk counties (Södermanland, Uppsala, Västmanland), with a total population 1.3 million, were identified through: contacting clinicians at all hospitals; surveillance of all suspected gastric cancer specimens at the departments of pathology; monthly double-checks with all regional cancer registers; and record linkages with the nation-wide Swedish cancer and death registries. All

*Received 24 November 1999*
*Revised 18 February 2000*
*Accepted 23 February 2000*

*Correspondence to:* AM Ekström cases resident in each county could be assigned to the correct population even if their disease was managed and reported outside of the study area, using the unique Swedish identification numbers (Lunde et al, 1980). By checking the regional and national cancer registries several years after the study period, cases missed through long delays in reporting could also be taken into account. Our thorough surveillance organization identified 100% of the cases recorded by the national Cancer Registry and an additional 30 cases missed by the Registry (Ekström et al, 1999).

### Classification

Clinical data, including a standardized account of the tumour centre in relation to anatomic landmarks, were prospectively obtained through special reports completed by clinicians during the entire study period. Hospital case records were scrutinized and all available (95%) histologic slides (both biopsy material and resection specimens for most patients) were reevaluated without reference to the initial report, by one pathologist. Each potential case was classified with regard to cancer status (gastric cancer yes/no), histologic type, and tumour site.

Intestinal differentiation was considered to be present when malignant cells formed definite glandular patterns (Lauren, 1965). Poorly differentiated cells, often signet-ring shaped and rarely forming glandular patterns, characterized the diffuse type (Lauren, 1965). The tumours were classified according to their predominant structure. Only when exhibiting approximately equal contributions of both types were they classified as being of mixed type. Haematoxylin-eosin and van Giesson, supplemented with Alcian blue-periodic acid Schiff (PAS) in biopsy specimens, were the stainings used. Cancer of the gastric cardia was defined as an adenocarcinomatous lesion with its centre located within 1 cm proximal and 2 cm distal to the gastro-oesophageal junction (Misumi et al, 1989). Tumours distal to this area were classified as non-cardia gastric cancers, while tumours located above were classified as oesophageal cancers. For 6% of cases the gastric site of the tumour could not be specified with certainty.

### Analyses

The incidence-rate denominators (person-years observed for each gender in each 5-year age group, county, and calendar year) were computed as the average of the population on January 1 and December 31 of the year in question. The incidence rates were directly standardized both to the Swedish (Epidemiology, 1997) and world populations (Parkin and Muir, 1992). We applied linear regression to test for trends in incidence rates over the study period and modelled the log of the incidence rates to estimate the annual percent change in these rates. A two-sample test for binomial proportions (Bland, 1987) was used for assessing differences in relative frequency between the two subgroups. We used univariate and multivariate logistic regression, estimated by the unconditional maximum likelihood method, to model the impact of gender, age, geographic risk area and year of diagnosis on the risk of developing intestinal-type adenocarcinoma compared to another histologic subtype (diffuse, mixed and unknown). We restricted the modelling to non-cardia tumours only. Odds ratios (OR) and 95% confidence intervals (CI) were used as measures of association.

### RESULTS

### Overall trends

A total of 1337 new cases of gastric adenocarcinoma were diagnosed in the study population from 1989–1994. There were 187 (14%) cardia cancers, 1069 (80%) non-cardia cancers and 81 (6%) gastric cancers where site could not be definitely established. Incidence rates (per 100 000 person-years), stratified by site, gender and calendar year are presented in Table 1.

Overall, we found a clear trend of declining incidence, with an estimated mean annual decrease of 6% (95% CI 3–8%). This decline was limited to adenocarcinomas located distal to the gastric cardia region: the incidence of cardia adenocarcinoma remained unchanged (Sweden age-standardized incidence 2.2 per 100 000 per year), while the incidence of non-cardia tumours fell

**Table 1** Incidence rates (IR) of gastric cancer per 100 000 person-years, 1989–1994, stratified by site, gender and calendar year

| | Case patients | | | Crude IR | | | Standardized IR[b] | | | World standardized IR[c] | | |
|---|---|---|---|---|---|---|---|---|---|---|---|---|
| | Total (*n*) | Male (*n*) | Female (*n*) | Total (*n*) | Male (*n*) | Female (*n*) | Total (*n*) | Male (*n*) | Female (*n*) | Total (*n*) | Male (*n*) | Female (*n*) |
| All sites[a] | | | | | | | | | | | | |
| 1989–1990 | 483 | 299 | 184 | 18.8 | 23.4 | 14.3 | 16.5 | 23.1 | 11.0 | 9.0 | 12.6 | 5.8 |
| 1991–1992 | 467 | 298 | 169 | 17.8 | 23.2 | 13.0 | 15.4 | 22.2 | 9.9 | 8.4 | 11.8 | 5.6 |
| 1993–1994 | 387 | 249 | 138 | 14.6 | 19.4 | 10.4 | 12.5 | 18.4 | 7.9 | 6.8 | 9.9 | 4.2 |
| 1989–1994 (entire period) | 1337 | 846 | 491 | 17.1 | 22.0 | 12.6 | 14.8 | 21.2 | 9.6 | 8.0 | 11.4 | 5.2 |
| Non-cardia | | | | | | | | | | | | |
| 1989–1990 | 410 | 245 | 165 | 15.9 | 19.1 | 12.8 | 13.9 | 18.9 | 9.8 | 7.4 | 10.1 | 5.2 |
| 1991–1992 | 375 | 232 | 143 | 14.3 | 17.7 | 10.9 | 12.4 | 17.3 | 8.5 | 6.7 | 9.1 | 4.9 |
| 1993–1994 | 284 | 174 | 110 | 10.7 | 13.1 | 8.3 | 9.1 | 12.7 | 6.3 | 4.9 | 6.8 | 3.3 |
| 1989–1994 (entire period) | 1069 | 651 | 418 | 13.7 | 16.6 | 10.7 | 11.8 | 16.3 | 8.2 | 6.3 | 8.7 | 4.4 |
| Cardia | | | | | | | | | | | | |
| 1989–1990 | 65 | 48 | 17 | 2.5 | 3.7 | 1.3 | 2.3 | 3.8 | 1.1 | 1.4 | 2.3 | 0.6 |
| 1991–1992 | 62 | 45 | 17 | 2.4 | 3.4 | 1.3 | 2.1 | 3.4 | 1.0 | 1.2 | 2.0 | 0.5 |
| 1993–1994 | 60 | 48 | 12 | 2.3 | 3.6 | 0.9 | 2.1 | 3.7 | 0.8 | 1.3 | 2.1 | 0.5 |
| 1989–1994 (entire period) | 187 | 141 | 46 | 2.4 | 3.6 | 1.2 | 2.2 | 3.6 | 0.9 | 1.3 | 2.1 | 0.5 |

[a]All sites include 81 gastric adenocarcinoma cases of unclassifiable subsite; [b]standardized to the Swedish population age distribution (Sweden 1970 census, Epidemiology, 1997); [c]standardized to the world population age distribution (Parkin and Muir, 1992)

 © 2000 Cancer Research Campaign

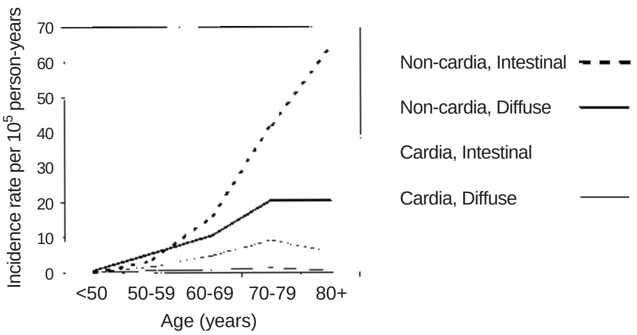

**Figure 1** Incidence rates of gastric adenocarcinoma per 100 000 person-years, for the entire study period (1989–1994), by histologic subtype, site and age by 9% (95% CI 6–12%) annually. The male:female ratios were 2:1 and 4:1 for non-cardia and cardia cancer respectively.

### Histologic type and site by gender and age

The histologic types were distributed as follows: 793 (59%) intestinal, 400 (30%) diffuse, 79 (6%) mixed type. In 65 cases (5%) the histologic type could not be determined due to a lack of tissue specimens. Incidence rates by histologic type, site, and age are presented in Figure 1. The relative distribution of histologic types and sites varied significantly by age. The diffuse type was significantly ($P$ < 0.0001) more common before age 50, comprising 100% of cases younger than 30 years and 56% among those aged 45–50. Table 2 shows the incidence rates of histologic types by site, gender and calendar year. The intestinal type predominated among men, where it accounted for 66% of all adenocarcinomas. In women, the corresponding figure was 48%. The male:female ratio was 3:1 and 4:1 for intestinal type adenocarcinomas of non-cardia and cardia location, respectively. The intestinal type was significantly ($P$ < 0.001) more predominant in the cardia region than in the rest of the stomach (75% vs 58%).

The diffuse type made up 40% of all adenocarcinomas in women, compared to less than 25% in men ($P$ < 0.0001). Hence, the male:female ratios for diffuse type tumours were only 1.2:1 and 2.5:1 for tumours of non-cardia and cardia location respectively.

### Secular trends by histologic type

Notwithstanding the short window of observation, we found highly significant and strikingly similar declines in both histologic subtypes. The intestinal type fell, on average, by 9% (95% CI 4–14%) annually, and the diffuse type by 8% (95% CI 3–15%). There was no similar trend for cancers originating in the cardia, of either the intestinal or diffuse type. When stratified by sex, the downward trend did not reach statistical significance among women for any site or subtype.

### Histologic type and geographic risk profile

The age-standardized annual incidence was 18.5 per 100 000 in the northern and 12.4 per 100 000 in the southern counties (Figure 2). The intestinal type was more predominant in the northern high-incidence counties: the ratio of the age-standardized intestinal to the diffuse tumour rates were 1.6:1 in the south and 2.2:1 in the north. However, this variation with latitude was confined to men. Up to 70% of all gastric adenocarcinomas among men in the high-risk counties were of the intestinal type, while diffuse type cancers constituted less than 20%. The decline in incidence was of similar magnitude in both geographic populations.

### Multivariate analyses

Using logistic regression we modelled the independent effects of gender, age, geographic risk profile and year of diagnosis on the probability of developing intestinal instead of any other histologic type of gastric adenocarcinoma (Table 3). We restricted this analysis to non-cardia cancers and therefore excluded 187 cases

**Table 2** Incidence rates (IR) of gastric cancer per 100 000 person-years, 1989–1994, by histologic subtype, gender and calendar year

| | Intestinal | | | | | | Diffuse | | | | | | Mixed | | | | | |
|---|---|---|---|---|---|---|---|---|---|---|---|---|---|---|---|---|---|---|
| | Crude | | | Standardized[b] | | | Crude | | | Standardized | | | Crude | | | Standardized | | |
| | Total | M[c] | F[d] | Total | M | F | Total | M | F | Total | M | F | Total | M | F | Total | M | F |
| All sites[a] | | | | | | | | | | | | | | | | | | |
| 1990 | 11.3 | 15.3 | 7.5 | 9.8 | 15.1 | 5.6 | 5.5 | 6.3 | 4.8 | 5.0 | 6.3 | 3.9 | 1.3 | 1.2 | 1.4 | 1.2 | 1.3 | 1.2 |
| 1992 | 10.2 | 14.7 | 5.8 | 8.7 | 14.5 | 4.0 | 5.6 | 5.5 | 5.8 | 5.1 | 5.3 | 5.1 | 1.1 | 1.6 | 0.7 | 0.9 | 1.4 | 0.6 |
| 1994 | 8.8 | 12.8 | 4.9 | 7.4 | 12.4 | 3.5 | 4.2 | 4.2 | 4.2 | 3.9 | 4.3 | 3.6 | 0.6 | 1.0 | 0.4 | 0.6 | 0.9 | 0.3 |
| 1994 (entire period) | 10.1 | 14.2 | 6.0 | 8.6 | 14.0 | 4.3 | 5.1 | 5.3 | 4.9 | 4.7 | 5.3 | 4.2 | 1.0 | 1.2 | 0.8 | 0.9 | 1.2 | 0.7 |
| Non-cardia | | | | | | | | | | | | | | | | | | |
| 1990 | 9.3 | 12.0 | 6.5 | 7.9 | 11.8 | 4.8 | 5.1 | 5.5 | 4.6 | 4.5 | 5.5 | 3.8 | 1.2 | 1.2 | 1.3 | 1.2 | 1.3 | 1.1 |
| 1992 | 8.1 | 11.5 | 4.7 | 6.8 | 11.4 | 3.1 | 4.9 | 4.4 | 5.5 | 4.5 | 4.3 | 4.8 | 0.9 | 1.2 | 0.6 | 0.8 | 1.1 | 0.5 |
| 1994 | 6.3 | 8.7 | 3.9 | 5.1 | 8.3 | 2.7 | 3.4 | 3.6 | 3.2 | 3.2 | 3.6 | 2.8 | 0.5 | 0.6 | 0.4 | 0.4 | 0.6 | 0.3 |
| 1994 (entire period) | 7.9 | 10.8 | 5.0 | 6.6 | 10.5 | 3.5 | 4.5 | 4.5 | 4.4 | 4.1 | 4.5 | 3.8 | 0.9 | 1.0 | 0.8 | 0.8 | 1.0 | 0.6 |
| Cardia | | | | | | | | | | | | | | | | | | |
| 1990 | 1.9 | 3.0 | 0.9 | 1.8 | 3.0 | 0.7 | 0.4 | 0.7 | 0.2 | 0.4 | 0.7 | 0.2 | 0.0 | 0.0 | 0.1 | 0.0 | 0.0 | 0.1 |
| 1992 | 1.6 | 2.3 | 1.0 | 1.5 | 2.3 | 0.8 | 0.4 | 0.7 | 0.2 | 0.4 | 0.7 | 0.1 | 0.1 | 0.2 | 0.0 | 0.1 | 0.2 | 0.0 |
| 1994 | 1.8 | 2.9 | 0.6 | 1.7 | 3.0 | 0.5 | 0.3 | 0.2 | 0.3 | 0.3 | 0.2 | 0.3 | 0.1 | 0.2 | 0.0 | 0.1 | 0.2 | 0.0 |
| 1994 (entire period) | 1.8 | 2.7 | 0.8 | 1.6 | 2.8 | 0.7 | 0.4 | 0.5 | 0.2 | 0.4 | 0.5 | 0.2 | 0.1 | 0.1 | 0.0 | 0.1 | 0.1 | 0.0 |

[a] All sites include 81 gastric adenocarcinoma cases of unclassifiable subsite; [b]standardized to the Swedish population age distribution (Sweden 1970 census, Epidemiology, 1997); [c]Male; [d]Female

*© 2000 Cancer Research Campaign*

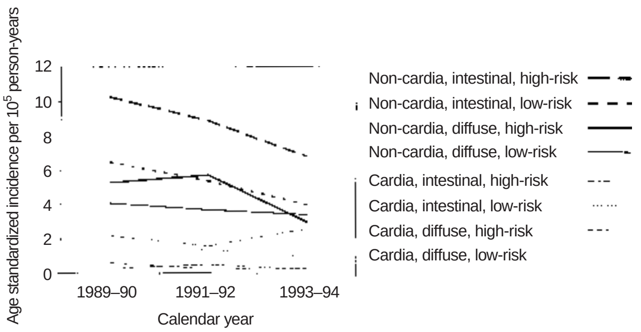

**Figure 2** Age-standardized incidence rates of gastric adenocarcinoma per 100 000 person-years, 1989–1994, by histologic subtype, site and geographic risk area (high vs low incidence)

with cardia tumours, and 20 cases with both histologic type and subsite unspecified. The marked deficit of intestinal type cancers in women relative to men remained (OR = 0.4, 95% CI 0.3–0.6) when controlling for the other factors. The proportion of the intestinal type increased rapidly with age ($P$ for trend < 0.05) and the relative risk was 8.5 of developing the intestinal type instead of another histologic subtype in the oldest age-group compared to the youngest (adjusted OR 8.5, 95% CI 4.4–16.3). Thus, gender and age both seemed to be independent, and important, risk factors for developing the intestinal type.

However, when we controlled for confounding by adding age, gender, and geographic risk profile to a multivariate model including year of diagnosis, the latter remained insignificant. Consequently, since year of diagnosis, i.e. secular trend, had no impact on the probability of developing the intestinal type instead of other gastric histologic subtypes, we could not confirm that the incidence of the intestinal type declined more rapidly than that of the diffuse type.

After adjustments, the predominance of the intestinal type in the high-incidence counties became statistically non-significant. There were no important interactions between the explanatory variables in our model.

### DISCUSSION

Contrary to the prevailing hypothesis, we found that the remarkably fast decrease in gastric cancers located distal to the cardia was not confined to the intestinal type. Indeed, the rate of the decline of the diffuse type was almost identical to that of the intestinal, strongly indicating that environmental rather than genetic factors determine the occurrence of both main histologic types.

Our results also confirm that the secular decline in gastric adenocarcinoma can be entirely attributed to a decrease of tumours located distal to the cardia region (Antonioli and Goldman, 1982; Correa and Chen, 1994; Howson et al, 1986), supporting the hypothesis that cardia and non-cardia cancers are distinct entities with diverging carcinogenesis. However, with a prospective, strictly uniform classification of site, we were unable to confirm the rising trend in cardia cancer reported by others (Blot et al, 1991; Correa and Chen, 1994; Hansson et al, 1993; Powell and McConkey, 1992; Yang and Davis, 1988); the incidence appeared stable during the 6 years of observation (1989–1994).

In our study, the risk of selection bias was reduced through a population-based design and comprehensive case ascertainment. Our data was even more complete (100% vs 98%) and correct than the Swedish Cancer Registry (Ekström et al, 1999) and changes in case identification over time were therefore unlikely. The blinded non-systematic case presentation to the pathologist would protect against gradual shifts in the histopathologic assessments, but random inter-observer variation cannot be excluded (Hansson et al, 1996). Though an unequivocal determination of the exact site of tumour origin can be difficult, misclassification was kept low through the standardized subsite reports, completed by the clinicians at the time of tumour detection. Only 6% of the cases remained unclassifiable to subsite of origin. Clinicians and pathology staff were also encouraged to report tumours bordering to adjacent sites, thus we would likely have detected any shift towards misclassification of cardia cancer as oesophageal.

Consistent with our findings of a decrease in both subtypes is the persistent predominance of the intestinal type of gastric adenocarcinoma in the USA despite a strong downward trend in gastric cancer and a now low total incidence. Sipponen et al pointed out that a decrease confined only to the intestinal type would have

**Table 3** Odds ratios (OR) and 95% confidence intervals (CI) for the risk of developing the intestinal type as opposed to other histologic subtypes of gastric adenocarcinoma[a]

| Explanatory variable | | Intestinal type Other subtypes | OR[b] | 95% CI | OR[c] | 95% CI |
|---|---|---|---|---|---|---|
| Gender | Male | 450/244 | 1.0 | – | 1.00 | – |
| | Female | 203/233 | 0.5 | 0.4–0.6 | 0.4 | 0.3–0.6 |
| Age (years) | ≤ 54 | 18/61 | 1.0 | – | 1.0 | – |
| | 55–59 | 19/34 | 1.9 | 0.9–4.1 | 2.0 | 0.9–4.3 |
| | 60–64 | 49/44 | 3.8 | 1.9–7.3 | 4.0 | 2.0–7.8 |
| | 65–69 | 87/55 | 5.4 | 2.9–10.0 | 5.4 | 2.9–10.3 |
| | 70–74 | 130/81 | 5.4 | 3.0–9.9 | 5.7 | 3.1–10.4 |
| | 75–79 | 143/93 | 5.2 | 2.9–9.4 | 5.8 | 3.2–10.6 |
| | 80–84 | 119/66 | 6.1 | 3.3–11.2 | 6.8 | 3.7–12.6 |
| | ≥85 | 88/43 | 6.9 | 3.7–13.2 | 8.5 | 4.4–16.3 |
| Geographic area | South | 314/263 | 1.0 | – | 1.00 | – |
| | North | 339/214 | 1.3 | 1.1–1.7 | 1.3 | 1.0–1.7 |
| Diagnostic year | 1989–1990 | 242/172 | 1.0 | – | 1.0 | – |
| | 1991–1992 | 224/172 | 0.9 | 0.7–1.2 | 0.9 | 0.7–1.2 |
| | 1993–1994 | 187/133 | 1.0 | 0.7–1.3 | 0.9 | 0.7–1.3 |

[a] Based on 1130 cases (cardia cancers excluded); [b]univariate logistic regression model (adjusted for age and gender); [c]multivariate logistic regression model including all variables in this table

 © 2000 Cancer Research Campaign caused its near complete depletion (Sipponen et al 1987). They also observed a significant decline for the diffuse type in Finland. Clear parallel declines of both histologic types among Connecticut women (Munoz and Connelly, 1971) correspond with a Swedish case series with no evidence of diverse secular trends for the two types over a 30-year period (1951–1981) (Lundegardh et al, 1991). Our results are, however, at odds with the decreasing ratios between the intestinal and diffuse types reported from the USA (Antonioli and Goldman, 1982), Norway (Munoz and Asvall, 1971), Germany (Mennicken et al, 1986), Italy (Amorosi et al, 1988), Finland (Lauren and Nevalainen, 1993), Iceland (Nikulasson et al, 1992), Japan (Hanai and Fujimoto, 1982) and Peru (Beteta et al, 1993). It is noteworthy that none of these studies were stratified for tumour site and most relied on hospital case series or registry data with a lower proportion of specimens classified histologically.

Six years may seem a short time in which to evaluate temporal patterns, but the clear consistent trends and the statistical precision makes chance alone a less likely explanation of our results. Although weak secular trends in cardia cancer may have escaped detection, we expected the incidence increase to be substantial due to previous reports (Antonioli and Goldman, 1982; Blot et al, 1991; Correa and Chen, 1994; Hansson et al, 1993; Powell and McConkey, 1992; Yang and Davis, 1988). Since we observed remarkably stable cardia cancer rates, random variation in incidence is unlikely to have masked any important trends. Instead, our findings hint that previous registry-based studies could be biased by shifts in classification practices. A recent validation study of cardia cancer diagnoses in the Swedish Cancer Register suggested that misclassification may be considerable (Ekström et al, 1999).

A declining occurrence of *H. pylori* infection in western populations (Haruma et al, 1997; Parsonnet et al, 1992; Roosendaal et al, 1997; Xia and Talley, 1997) coincides with a decreasing incidence of gastric cancer and supports a causal pathway between *H. pylori*-activated inflammation and the intestinal type of adenocarcinoma, via atrophy, intestinal metaplasia and dysplasia (Correa and Shiao, 1994). But since the peri-tumour zone of most diffuse cancers lack these long-lasting intermediate steps, the importance of a preceding *H. pylori* infection in this subtype may be questioned. It has been suggested that *H. pylori*-induced chronic gastritis, involving an active renewal of gastric epithelium sensitive to oxidative carcinogenes, could be an early common starting-point in the genesis of both histologic types (Solcia et al, 1996). If genes primarily responsible for cell proliferation and differentiation are damaged, intestinal type cancer may then develop via the intermediate steps outlined above. Alternatively, if the early *H. pylori*-activated carcinogenic process affects genes involved in cell adhesion and basal membrane integrity, the secretory mucosal function would be retained (signet-ring cells), while prerequisites are formed for the invasiveness characterizing diffuse adenocarcinomas (Solcia et al, 1996).

The inconsistent findings regarding *H. pylori*'s role in the genesis of the two histologic types (Solcia et al, 1996; Hansson et al, 1995; Parsonnet et al, 1993; Talley et al, 1991) may, apart from lack of statistical power, also be explained by retrospective studies of a bacteria which often spontaneously disappears from the mucosa during malignant transformation but before diagnosis of the tumour.

In conclusion, our observations suggest that the notion of an environmentally predetermined intestinal type accompanied by a genetically predetermined diffuse type (Correa and Shiao, 1994; Howson et al, 1986; Lauren and Nevalainen, 1993; Stemmermann and Brown, 1974; Lehtola, 1981) may need to be modified. Environmental factors, including *H. pylori*, could be as important for diffuse adenocarcinomas as they are for the intestinal type. However, the marked gender differences in subtype incidence as well as secular trends, despite similar *H. pylori* sero-prevalence among men and women, indicate that other factors, possibly modifying the effect of *H. pylori*, are also important.

### ACKNOWLEDGEMENTS

We thank Lotti Barlow, Centre for Epidemiology, The National Board of Health and Welfare, for providing us with population data. This work was supported by a grant from the National Cancer Institute (R01 CA 50959).

### REFERENCES

Amorosi A, Bianchi S, Buiatti E, Cipriani F, Palli D and Zampi G (1988) Gastric cancer in a high-risk area in Italy. Histopathologic patterns according to Lauren's classification. *Cancer* **62**: 2191–2196

Antonioli DA and Goldman H (1982) Changes in the location and type of gastric adenocarcinoma. *Cancer* **50**: 775–781

Beteta O, Lozano R, Monge E, Yoza M and Cavero J (1993) Change in the histologic type of gastric carcinoma (letter, comment). *Am J Gastroenterol* **88**: 787–788

Bland M (1987) *An introduction to medical statistics*. Oxford Medical Publications, Oxford University Press: Oxford & New York

Blot WJ, Devesa SS, Kneller RW and Fraumeni JF, Jr (1991) Rising incidence of adenocarcinoma of the esophagus and gastric cardia. *JAMA* **265**: 1287–1289

Coleman MP, Esteve J, Damiecki P, Arslan A and Renard H (1993) Trends in cancer incidence and mortality. *IARC Sci Publ* **121**: 1–806

Correa P (1995) *Helicobacter pylori* and gastric carcinogenesis. *Am J Surg Pathol* **19**: S37–43.

Correa P and Chen VW (1994) Gastric cancer. *Cancer Surv* **20**: 55–76

Correa P and Shiao YH (1994) Phenotypic and genotypic events in gastric carcinogenesis. *Cancer Res* **54**: 1941s–1943s

Ekström AM, Signorello LB, Hansson LE, Bergstrom R, Lindgren A, Nyren O (1999) Evaluating gastric cancer misclassification: a potential explanation for the rise in cardia cancer incidence. *J Natl Cancer Inst* **91**: 786–790

Epidemiology (1997) Cancer Incidence in Sweden 1994. In *Statistics – Health and Diseases*, Vol. 2. The National Board of Health and Welfare: Uppsala

Hanai A and Fujimoto I (1982) Cancer incidence in Japan in 1975 and changes of epidemiological features for cancer in Osaka. *National Cancer Institute Monographs* **62**: 3–7

Hansson LE, Lindgren A and Nyren O (1996) Can endoscopic biopsy specimens be used for reliable Lauren classification of gastric cancer? *Scand J Gastroenterol* **31**: 711–715

Hansson LE, Sparen P and Nyren O (1993) Increasing incidence of carcinoma of the gastric cardia in Sweden from 1970 to 1985. *Br J Surg* **80**: 374–377

Hansson LR, Engstrand L, Nyren O and Lindgren A (1995) Prevalence of *Helicobacter pylori* infection in subtypes of gastric cancer. *Gastroenterology* **109**: 885–888

Haruma K, Okamoto S, Kawaguchi H, Gotoh T, Kamada T, Yoshihara M, Sumii K and Kajiyama G (1997) Reduced incidence of *Helicobacter pylori* infection in young Japanese persons between the 1970s and the 1990s. *J Clin Gastroenterol* **25**: 583–586

Howson CP, Hiyama T and Wynder EL (1986) The decline in gastric cancer: epidemiology of an unplanned triumph. *Epidemiol Rev* **8**: 1–27

Lauren PA (1965) The two main histological types of gastric carcinoma: Diffuse and so-called intestinal type carcinoma. *Acta Pathologica Microbiologica Scandivica* **64**: 31–49

Lauren PA and Nevalainen TJ (1993) Epidemiology of intestinal and diffuse types of gastric carcinoma. A time-trend study in Finland with comparison between studies from high- and low-risk areas. *Cancer* **71**: 2926–2933

Lehtola J (1981) Family behaviour of gastric carcinoma. *Annals of Clinical Research* **13**: 144–148

Lunde AS, Lundeborg S, Lettenstrom GS, Thygesen L and Huebner J (1980) The person-number systems of Sweden, Norway, Denmark, and Israel. *Vital Health Stat* **2**: 1–59

Lundegardh G, Lindgren A, Rohul A, Nyren O, Hansson LE, Bergstrom R and Adami HO (1991) Intestinal and diffuse types of gastric cancer: secular trends in Sweden since 1951. *Br J Cancer* **64**: 1182–1186

Mennicken C, Bohrer MH, Jung M and Manegold BC (1986) [New aspects of early gastric carcinoma]. *Dtsch Med Wochenschr* **111**: 255–258

Misumi A, Murakami A, Harada K Baba K and Akagi M (1989) Definition of carcinoma of the gastric cardia. *Langenbecks Arch Chir* **374**: 221–226

Munoz N and Asvall J (1971) Time trends of intestinal and diffuse types of gastric cancer in Norway. *Int J Cancer* **8**: 144–157

Munoz N and Connelly R (1971) Time trends of intestinal and diffuse types of gastric cancer in the United States. *Int J Cancer* **8**: 158–164

Munoz N and Franceschi S (1997) Epidemiology of gastric cancer and perspectives for prevention. *Salud Publica Mex* **39**: 318–130

Nikulasson S, Hallgrimsson J, Tulinius H, Sigvaldason H and Olafsdottir G (1992) Tumours in Iceland. 16. Malignant tumours of the stomach. Histological classification and description of epidemiological changes in a high-risk population during 30 years. *Apmis* **100**: 930–941

Parkin DM and Muir CS (1992) Cancer incidence in five continents. Comparability and quality of data. *IARC Sci Publ* **120**: 45–173

Parkin DM, Pisani P and Ferlay J (1993) Estimates of the worldwide incidence of eighteen major cancers in 1985. *Int J Cancer* **54**: 594–606

Parsonnet J, Blaser MJ, Perez-Perez GI, Hargrett-Bean N and Tauxe RV (1992) Symptoms and risk factors of *Helicobacter pylori* infection in a cohort of epidemiologists. *Gastroenterology* **102**: 41–46

Parsonnet J, Samloff IM, Nelson LM, Orentreich N, Vogelman JH and Friedman GD (1993) *Helicobacter pylori*, pepsinogen, and risk for gastric adenocarcinoma. *Cancer Epidemiological Biomarkers Preview* **2**: 461–466

Powell J and McConkey CC (1992) The rising trend in oesophageal adenocarcinoma and gastric cardia. *Eur J Cancer Prev* **1**: 265–269

Roosendaal R, Kuipers EJ, Buitenwerf J, van Uffelen C, Meuwissen SG, van Kamp GJ, and Vandenbroucke-Grauls CM (1997) *Helicobacter pylori* and the birth cohort effect: evidence of a continuous decrease of infection rates in childhood. *Am J Gastroenterol* **92**: 1480–1482

Sipponen P, Jarvi O, Kekki M and Siurala M (1987) Decreased incidences of intestinal and diffuse types of gastric carcinoma in Finland during a 20-year period. *Scand J Gastroenterol* **22**: 865–871

Solcia E, Fiocca R, Luinetti O, Villani L, Padovan L, Calistri D, Ranzani GN, Chiaravalli A and Capella C (1996) Intestinal and diffuse gastric cancers arise in a different background of *Helicobacter pylori* gastritis through different gene involvement. *Am J Surg Pathol* **20**: S8–

Stemmermann GN and Brown C (1974) A survival study of intestinal and diffuse types of gastric carcinoma. *Cancer* **33**: 1190–1195

Talley NJ, Zinsmeister AR, Weaver A, DiMagno EP, Carpenter HA, Perez-Perez GI and Blaser MJ (1991) Gastric adenocarcinoma and *Helicobacter pylori* infection. *J Natl Cancer Inst* **83**: 1734–1739

Xia HH and Talley NJ (1997) Natural acquisition and spontaneous elimination of Helicobacter pylori infection: clinical implications. *Am J Gastroenterol* **92**: 1780–1787

Yang PC and Davis S (1988) Epidemiological characteristics of adenocarcinoma of the gastric cardia and distal stomach in the United States, 1973–1982. *Int J Epidemiol* **17**: 293–297

*© 2000 Cancer Research Campaign*
